# Challenges in Navigating the Health Care System: Development of an Instrument Measuring Navigation Health Literacy

**DOI:** 10.3390/ijerph17165731

**Published:** 2020-08-08

**Authors:** Lennert Griese, Eva-Maria Berens, Peter Nowak, Jürgen M. Pelikan, Doris Schaeffer

**Affiliations:** 1 School of Public Health, Interdisciplinary Centre for Health Literacy Research [ICHL], Bielefeld University, 33615 Bielefeld, Germany; eva-maria.berens@uni-bielefeld.de (E.-M.B.); doris.schaeffer@uni-bielefeld.de (D.S.); 2 Department Health and Society, The Austrian Public Health Institute [GÖG], 1010 Vienna, Austria; peter.nowak@goeg.at; 3 WHO-CC Health Promotion in Hospitals and Health Care, The Austrian Public Health Institute [GÖG], 1010 Vienna, Austria; juergen.pelikan@goeg.at

**Keywords:** health literacy, hls-eu, navigation, orientation, health care system, instrument development, questionnaire, validation

## Abstract

Due to their rapid expansion and complexity, it is increasingly difficult for patients to orient themselves in health care systems. Therefore, patients require a high degree of health literacy, or more precisely, navigation health literacy (HL-NAV). The actual extent of HL-NAV of patients and citizens is still largely unknown due to the lack of adequate measurement instruments. Thus, within the new international Health Literacy Population Survey 2019 (HLS_19_), one aim was to develop a suitable instrument for measuring HL-NAV in the HLS_19_ the HL-NAV-HLS19. The item development was conducted by an international working group within the HLS_19_ Consortium led by the first and last authors. Methodologically, it is based on a scoping literature review, development of a conceptual framework for HL-NAV, and first item formation, as well as an evaluation by experts, stakeholders, focus groups, pre-test interviews, and continuously feedback from the HLS_19_ Consortium. HL-NAV was defined as the ability to access, understand, appraise, and apply information on navigational issues, drawing on ten selected publications and the health literacy definition of the HLS-EU Consortium. Main tasks of HL-NAV at the system, organization, and interaction level were identified, to which first related items were assigned. Based on the feedback from experts, the focus group discussions, and the HLS_19_ Consortium, the instrument was slightly revised. Finally, twelve items proved to be feasible in the pre-test. The instrument will be used for the first time in the HLS_19_ survey and will provide first data on HL-NAV in general populations for the countries participating in HLS_19_. It is suited for cross-country comparisons and monitoring, as well as for intervention development. However, the instrument should be translated into and validated in further languages and countries for population samples.

## 1. Introduction

Health care systems in many countries have become more and more complex and confusing as a result of increasing expansion and specialization over the past decades [1,2,3,4,5]. Thus, the demands on patients and users to orient within and navigate health care systems are increasing as well [6]. Patients are required to identify an adequate entry point to the health care system, to orientate themselves within a multitude of organizations, to maneuver through the system, and to find the right place for their own problems [7,8,9,10]. Similarly, it is important to find one’s way around the organization and to interact and communicate in such a way that the questions and problems encountered are adequately answered and informed decision making for health care is possible [11]. Not all patients and users are able to meet such navigation requirements, leading to disorientation, futile and stressful searches, uncertainty, and discontinuities in health care [12,13]. Studies indicate that this particularly affects people with low health literacy [14,15,16,17]. They have more problems in finding their way within the health care system and in dealing with the often-complicated information.

Following the definition of the HLS-EU Consortium [18] (p. 3) comprehensive health literacy can be understood as the “knowledge, motivation and competences, to access, understand, appraise, and apply health information in order to make judgments and take decisions in everyday life concerning health care, disease prevention and health promotion to maintain or improve quality of life during the life course”. When it comes to using and navigating the health care system, a specific form of comprehensive health literacy is required, i.e., navigation health literacy (HL-NAV). HL-NAV includes being able to handle information in such a way that it is possible to navigate the health care system without any difficulty and to “find the right care at the right time in the right place” [19] (p. 14). Whether this is successful depends on the one hand on the abilities of the individual, as described by Sørensen et al. [18], and on the other hand on the demands and complexity of the system that must be navigated [20].

While the empirical knowledge about health literacy in populations is increasing, the topic of navigation in health care has received relatively little specific attention. When navigation problems are addressed, this is mostly done with regard to barriers or coordination deficits in health care, the management of (chronic) illness [21,22,23,24], as well as in terms of the objectives of compensatory intervention concepts, such as case or care management and patient navigation [25,26,27]. The ability to handle information in order to find one’s way around the health care system, is still rarely studied. Although the work by Rima Rudd and colleagues [28] took the topic quite early on the agenda, comprehensive analyses of HL-NAV are still missing until today. This is why the issue was integrated as an optional package into the International Health Literacy Population Survey 2019 (HLS_19_). The HLS_19_ project is part of the M-POHL Network, which was established under the umbrella of WHO Europe’s Health Information Initiative (EHII) with the adoption of the Vienna Statement on the Measurement of Health Literacy in Europe [29]. The survey aims at measuring health literacy in different countries based on the concept and the instruments of HLS-EU [30]. HLS_19_ also includes newly developed supplementary packages. HL-NAV is one of them. For this purpose, a new instrument for measuring HL-NAV was developed for cross-country comparisons and monitoring, as well as for intervention development. The steps taken to achieve this are described in this article.

## 2. Materials and Methods

**Figure 1:**
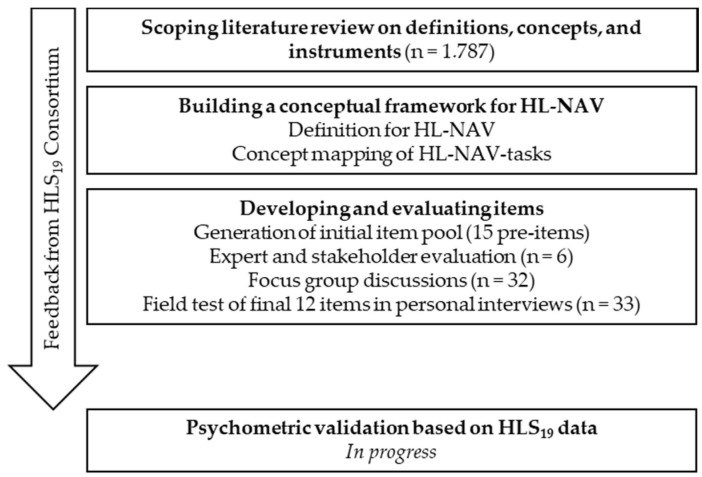
Development process of the instrument to measure HL-NAV.

The procedure followed standards for the development of measurement instruments in social science survey research [31]. The entire process is outlined in Figure 1. The steps undertaken were part of the preparation for the HLS_19_ and were carried out within the M-POHL Network. A working group on measuring HL-NAV was established, consisting of seven international experts from countries participating in the HLS_19_ (Germany, Austria, Switzerland, Norway, Portugal, and Czech Republic) and led by the first and last authors. Representatives from HLS_19_ countries interested in developing the package HL-NAV were able to join the working group at the early stage of HLS_19_ preparations.

The project was conducted in accordance with the Declaration of Helsinki; focus group discussions were approved by the Ethics Committee of Bielefeld University (application number EUB 2019-247).

### 2.1. Scoping Literature Review

**Figure A1:**
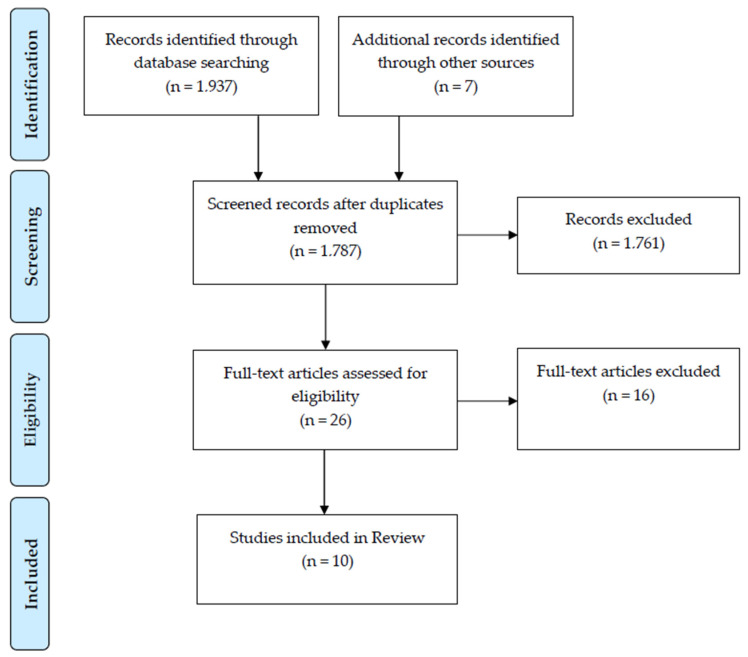
PRISMA flow chart for search process.

At first, a scoping literature review using Arksey and O’Malley’s [32] approach was performed to obtain an overview of existing definitions, concepts, and instruments in the field of navigation with a special focus on health literacy. The underlying research question was: Which approaches exist to conceptualize and measure navigation in the health care system and what role does health literacy play? The search was carried out in PubMed and CINAHL from November 2018 to January 2019. It was extended by a hand search. A combination of the keywords (health services, health services accessibility, health organization, health care system, health care, health care literacy, navigation, orientation, patient experience, patient preference, patient perception, patients’ view, family perspective, consumer, user, caregiver, patients) was used. The detailed procedure, inclusion and exclusion criteria are listed in the Table A1 and Figure A1.

### 2.2. Conceptual Framework for HL-NAV

In a second step, a working definition of HL-NAV was developed that addressed the underlying understanding and principles of health literacy [18,33] as well as the findings from the reviewed literature. Afterwards, concept mapping of the main HL-NAV tasks was carried out. Concrete information tasks, which patients and users must perform while navigating health care systems were derived from literature, discussed, supplemented by the research team, embedded in the context of information processing, and revised throughout the development process.

### 2.3. Item Development and Evaluation

Finally, items matching these tasks have been developed. The wording and response scale of the European Health Literacy Survey Questionnaire (HLS-EU-Q) was applied for item development. The HLS-EU-Q captures the subjective difficulties in accessing, understanding, appraising, and applying health information in three domains: health care, disease prevention, and health promotion and therefore assesses “comprehensive” health literacy [30] (p. 19). Existing items from instruments identified by the literature review (step 1) and from instruments referring to single HL-NAV tasks [34,35,36] were adjusted and reformulated to fit into this format of operationalization. In addition, new items were formulated for tasks where no items could be retrieved from the existing literature.

The initial item pool was evaluated with regard to its importance by six German speaking experts from health literacy research (n = 2), and health services research (n = 1), also by stakeholders in health care (n = 3). The Content Validity Index for Items (I-CVI) and Scales (S-CVI) was applied to quantify content validity—a method widely used to establish and quantify content validity in diverse fields [37]. Experts were requested to give written feedback on the proposed items by rating how well each item reflected the working definition of HL-NAV on a 4-point-Lickert scale from “1 = not relevant” to “4 = highly relevant” as requested by Davis [38]. The I-CVI was computed as the proportion of experts giving a rating of either 3 or 4 [37]. The S-CVI was calculated by the averaging calculation approach (S-CVI/Ave) using the average of the I-CVIs for all items on the scale. Furthermore, experts were asked whether each item is comprehensible and clearly formulated and whether they have any recommendations and subjects which had thus far not been considered.

In a next step, items were discussed in four different focus groups—a procedure which is frequently used in initial item development [39,40]. The aim was to gain feedback on the clarity and interpreted content of each item. Furthermore, additional recommendations provided by the participants were noted. The focus groups were organized by a professional institute. Two focus group discussions with eight participants each took place in an urban region, and two were carried out in a more rural region. The participants had different socio-demographic backgrounds. In concrete, for 3/4 focus groups a different selection criterion was used (chronic illness, low level of education, unspecified migration background). In addition, one group was conducted without any predefined criteria. Further overarching criteria were a balanced gender ratio and a balanced age distribution as possible. Participants had to be over 18 years. Each focus group was moderated by two researchers.

The final instrument to be used for Germany was pre-tested in thirty-three personal face-to-face interviews. These interviews were conducted by interviewers from a professional research institute. The interviewees were recruited from the general population aged 18 and over. A balanced distribution of gender, age, and education was sought here. In parallel, a pre-test under field-conditions was performed by twelve staff members of the institute, half of them as interviews and the other half as expert reports. The results of the two pre-test modules were collected and evaluated together with the research team.

During the entire development process, the current item status was constantly translated back and forth between German and English in order to obtain feedback from the working group members. The methodological approach and the status of the instrument were also presented and discussed at two M-POHL meetings in Dublin and Berlin within the whole HLS_19_ Consortium. To ensure that the English translation meets the initial intention, the final English version was also translated back by an external survey agency into German. A final language check for the German version was conducted with two researchers from Austria and Switzerland with regard to translation and country/system-specific wording.

## 3. Results

### 3.1. Scoping Literature Review

In total, the literature research identified 1787 publications, of which 1761 were not included after titles and abstracts were screened. Of the remaining twenty-six, a total of ten publications were classified as relevant after full-text screening because they either provided an understanding of navigation (health literacy) or/and offered a quantitative instrument related to the topic. The results are shown in Table 1.

The studies considered can be distinguished with regard to the conceptualization of navigation in the health care system and the addressed skills and competencies. For the latter there are only a few studies addressing health literacy in terms of navigation [28,41,42]. Most studies tend to focus more on the skills and processes required to find one’s way around the health care system [9,43,44,45,46]. In these studies, navigation or—in the words of Gui and colleagues [46] (p. 2)—“navigational competence” is primarily conceptualized as the knowledge and skills needed to find and use suitable services in the health care system. These skills are linked to information and experience as a source of knowledge and were conceptualized and examined as a potential outcome of health literacy [43,44,45].

In contrast, a clear connection to health literacy is made by Rima Rudd and colleagues [28,41]. In an exploratory study, entitled Navigating Hospitals, Rudd showed that health organizations represent “literate environments” [41] (p. 23), whose navigation requires specific health literacy skills. These tasks assigned to the individual are strongly dependent on the quality of the information available for navigation in organizations. If it is insufficient, the demands placed on the individual increase. The same applies to the system level: the more complex health care systems are, the more demanding is the navigation and the more demanding are the arising HL-NAV tasks [28].

There are also some studies focusing on the skills needed to interact, communicate, and negotiate with health organizations and health professionals in order to participate in health care decisions or to access needed services and treatments [47,48]. Although there is no clear reference to health literacy in these studies, it can be assumed that the concepts examined here include aspects of communicative health literacy [49].

With regard to the concept of navigation, it can be stated that there is no common understanding of what is meant by navigation concerning the health care system. Navigation is mostly defined as the ability to orientate oneself and find one’s way around a topographical area in order to find, access, and use a desired health service. Therefore, it takes place either on system level, between, or within health care organizations [9,28,32,41,44,45,46]. As noted above, in some studies, the term navigation is also used in the context of interaction and communication [47,48].

With regard to the measurement of HL-NAV, only two studies provided a quantitative instrument [28,42] addressing health literacy tasks in terms of navigation: Within the subscale *Systems Navigation* of the *Health Activities Literacy Scale (HALS)*, an assessment of literacy skills is included, which refers to bureaucratic procedures in order to get access to services or make use of rights and responsibilities as a user of the health care system [28]. A more comprehensive understanding of health literacy is pursued with the *Health Literacy Questionnaire (HLQ)* [42], in which the ability to find out about services and supports and to advocate on the own behalf in the health care system is assessed with one subscale. Even though a clear reference to health literacy is made with the scale’s location in the HLQ, the link to information processing on item level is rather low and the act of dealing with information is only partially assessed.

Overall, there are only a few studies emphasizing the importance of HL-NAV in the health care system. However, no conceptual understanding or instrument was found that uses a broader definition of HL-NAV, including the four steps of information processing on the macro, meso, and micro level.

### 3.2. Conceptual Framework for HL-NAV

Based on the literature on navigation (health literacy) and the current health literacy definition as put forward by the HLS-EU Consortium [18], the following working definition for HL-NAV was developed in order to create a consistent conceptual foundation for further item development:

“Navigation Health Literacy (HL-NAV) refers to people’s knowledge, motivation and skills to access, understand, appraise and apply the information and communication in various forms necessary for navigating health care systems and services adequately to get the most suitable health care for oneself or related persons”.

**Figure 2:**
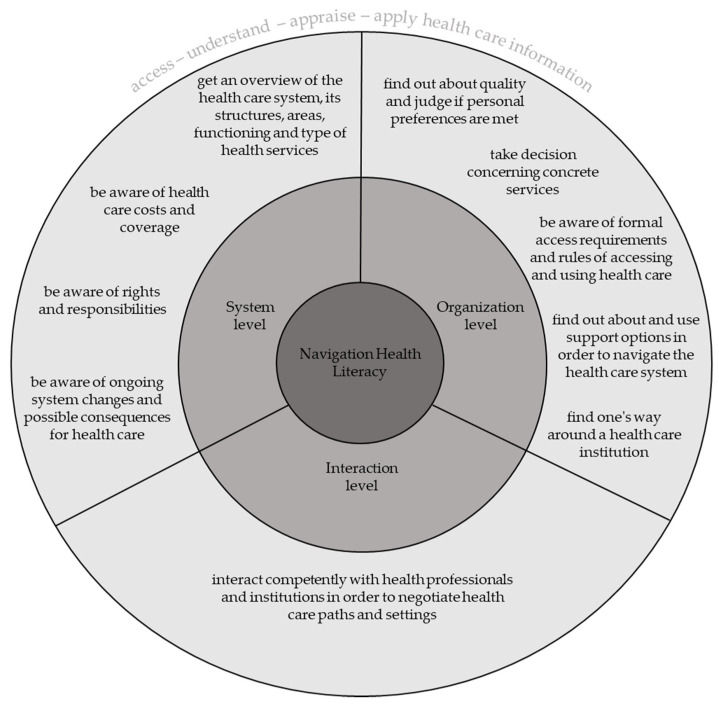
The main HL-NAV tasks.

Figure 2 gives an overview of ten different kinds of tasks involved in navigating the health care system adequately on the interaction (micro), the organization (meso), and the system (macro) level, which emerged from the mapping procedure. Three of these tasks emerged from expert/stakeholder feedback and item discussions in the HLS_19_ Consortium and were added during the evaluation phase.

Since navigation health literacy, like comprehensive general health literacy, is to be understood as a relational concept [20], the actual navigation health literacy of a person in a specific situation depends on her/his personal navigation health literacy as well as on the complexity and demands on navigation by the health care system in question, especially on the quality and forms of information and communication offered to support navigating a health care system. Therefore, actual HL-NAV of people can best be improved by making these organizational qualities more userfriendly. However, to do this effectively, it is necessary to know and measure where people have personal difficulties in navigating their health care system.

Following the definition, HL-NAV in our study mainly refers to the health literacy domain “health care” in the conceptual model of the HLS-EU Consortium [30], whereby reference should be made here to the term *care*, which is to be understood in distinction to the term *cure* [50]. While cure is primarily intended to address clinical aspects, mainly related to acute illness and intervention (e.g., therapy or treatment), the term care applies to the social organization and coordination of necessary services and processes within the whole health care system [51]. Care, as mentioned in the HL-NAV definition, encompasses all areas of health care provided in society by myriad other institutions and across system boundaries, including social, mental, nursing, or rehabilitative health care services.

Moreover, HL-NAV is conceptualized on three different levels: on the *system level*, on the *organization level*, and on the *interaction level*. The system level primarily addresses the act of orientation and of obtaining an overview of the health care system, its structures, interrelationships, and functioning (e.g., how is the health system organized, how does it function and work). At the organizational level the patients and users have to process information as a perquisite for joint decisions on using particular health services in the most adequate way (e.g., which organization functions in which way, who is the right contact person in there, and what are the rules to use it). In line with Nutbeam’s [49] considerations, the interactional/communicative level stresses the necessity of assuming a more active role in processing information about health. With regard to navigation, this includes the articulation of preferences as well as the ability to obtain information from health services and health professionals in order to participate in decisions about and negotiate and plan further health care use (e.g., how must patients behave and interact, how can they communicate their own problems in such a way that a workable solution can be jointly discussed and agreed upon?).

### 3.3. Item Development and Evaluation

Fifteen initial items were developed to reflect the main HL-NAV tasks and were used for testing in expert and focus group evaluation. During the evaluation phase, three new items were added according to the extension of the concept. With regard to the HLS-EU-Q, a four-point Likert scale with the response categories 1 = very difficult, 2 = difficult, 3 = easy, 4 = very easy was chosen with the objective to calculate an index referring to the HLS-EU procedure [30].

The range of content validity indices from *the expert and stakeholder evaluation* are shown in Table 3. The content validity index for Scale (S-CVI/Ave) reached a value of 0.90 and thus can be considered acceptable. Experts assessed a total of twelve of the fifteen items as relevant for operationalizing the construct (I-CVI ≥ 0.83). Items 1, 9, and 15 only achieved a point value of 0.67; as a result, these items were substantially revised or replaced by a new item. Furthermore, experts noted that certain items were formulated too similarly; therefore, these items were combined. Further recommendations by the experts were to: (1) use consistent terminology throughout the questionnaire (this applies in particular to frequently used terms such as “health care system”, “health care services”, “health care institution”), (2) create less complex information tasks and instead create specific situations in which information on health care is used, (3) ensure clear language and avoid expert terminology: clarify with explanations, (4) do not formulate the items in a too complex fashion, and keep in mind that they will be asked in an extensive questionnaire, (5) assign the four steps of information processing logically to the used situations.

A total of thirty-two persons participated in four *focus groups*. In each discussion, a different population group was addressed to ensure that at least eight participants with chronic illness, low education, and migration background were included. In addition, eight participants without any specific background took part. The characteristics of the participants are outlined in Table 2. Overall, the majority of items were interpreted by participants as intended by the experts. The term “user” was criticized by serval participants (initial item 10: *understand your rights as a user of the health care system)*, and some participants associated it more with the use of online services or digital applications. As a result, the participants’ suggestion to use the terminology “patient or user of the health care system” was adopted for clarification. This also applies to the term “health care institution”, which could not be clearly assigned by some participants. In line with the expert feedback, it was recommended to supplement those items with examples and define recurring terms at the beginning of the questionnaire with help of an introduction. This overall request was taken into account by adding examples to selected items as well as by giving an introduction as follows: *“Now we would like to know how easy it is to inform yourself on finding your way around the health care system. It does not matter whether you use information for yourself or for someone else. By health service we typically mean a doctor, specialist, hospital, nursing home, rehabilitation or mental health facility”.* In addition, some of the respondents noted that the wording “professional help” (initial item 3: *find information about where to get professional help when you are ill?)* could also be misunderstood, because it is used synonymously in the context of seeking mental health services in Germany; therefore, the term was no longer used. Furthermore, there was also a lively discussion of the initial item 13 (*to find one’s way around a health care institution).* While the item was mainly understood as intended by people with chronic illness, people who did not come, or only rarely came, into contact with complex care facilities—such as a hospital or a nursing home—had greater difficulties in understanding the idea of the item. Here, the respondents argued for summarizing the aspect under item 14, which was assessed as more understandable and relevant (initial Item 14: *to locate the right contact person for your concern within a health care institution*).

After incorporating the recommendations from the expert and focus group evaluations, twelve items measuring the perceived difficulties in accessing (three items), understanding (three items), appraising (three items), and applying (three items) health information in the context of navigation were field-tested. The characteristics of the pre-test sample are also outlined in Table 2. Six items were assigned to the system, six to the organizational, and one to the interactional level.

A main recommendation from the field-test was to clarify the focus of the overall instrument. Thus, it was not clear whether (a) a personal rating (an estimation of how easy or difficult it is to assess something for oneself), (b) a general rating (how easy or difficult it is to assess something in general), or (c) a personal experience (how easy or difficult it is or has been) was required. To clarify the researchers’ intention of a general assessment, the following formulation was used at the beginning of the questionnaire: “*On a scale from very easy to very difficult, how easy would you say it is to*”. Apart from that, the field test did not result in changes to the instrument. The overall revisions were made, and the exact wording of each final item is presented in Table 3.

## 4. Discussion

The fragmentation, complexity, and non-transparency of health care systems and the resulting problems have long been discussed and criticized [2,22,52,53,54,55,56]. However, the consequences for patients are far less often taken into account. However, patients are the ones to suffer the most from complicated und fragmented health care systems because such systems cause supply deficits and impede easy navigation and use of health services. The same applies to information required for navigation: it is also not easy to find, often difficult to understand, and can only be used to a limited extent for orientation and health services use. Therefore, it is all the more important to analyze the difficulties in navigation, and even more so the HL-NAV, necessary to better deal with it, in more detail. So far—as the literature analysis and comparable reviews on health literacy have shown [57]—this has been done only very cautiously.

The development of the instrument described here is an attempt to contribute to this aim. With the use of the instrument in the HLS_19_, data on HL-NAV in several countries will be collected for the first time. This will provide important comparative insights for intervention development and further research. In terms of content, the instrument focusses on the use of health services by patients and the role of health literacy in this context. Conceptually, it is based on a multidimensional understanding of health literacy that includes functional skills but goes beyond these and includes interactive and critical skills that are relevant for the context of navigation. It therefore gives equal weight to the information dimensions (accessing, understanding, appraising, and applying) regarded as central to health literacy and anchored in the underlying framework [18]. Furthermore, health literacy is understood as a relational construct [20]. By measuring the subjective difficulties in dealing with health information, a relational understanding of health literacy is taken into account—as has been the case in the comprehensive instrument of the HLS-EU. At the same time, the developed instrument goes beyond a clinical perspective and allows an assessment of HL-NAV in different populations. Therefore, it meets the demand for an increased operationalization of health literacy from a public health perspective [33]. A further strength of the tool is that its underlying concept maps HL-NAV at the system, organization, and interaction levels. Previous instruments mostly lack these detailed perspectives.

For the validation of items by focus groups or cognitive interviews, the literature discusses the possibility of involving either laypersons or people with background knowledge [58,59]. The focus groups in this study primarily involved people with no proven knowledge of the health care system, even though there was a high proportion of the chronically ill, who probably had greater system experience and probably also knowledge.

The validity of the content, as assessed by the CVI, allowed a relatively easy estimation of the relevance of the single items and the suitability of the entire instrument. The number of 6 experts/stakeholder in this study can be considered as appropriate for ensuring content validity [37]. If there are six or more judges, the I-CVI should not be lower than 0.78. For the S-CVI, it is indicated that a value of 80 or higher is acceptable [38,60,61]. The CVI for the entire item pool (S-CVI) fulfils the requirements highlighted in the relevant literature. The CVI for individual items (I-CVI) allowed a targeted revision of the instrument. However, at this point, a quantifiable assessment of the content validity could only be made for the initial item pool, since the results of the validation and additional feedback in the development process led to further adjustments and additions to the instrument. Nevertheless, the substantive focus has been maintained, so that it is assumed that the instrument complies with the criteria of content validity applied here.

It is also emphasized that the instrument was developed involving international experts, important stakeholders, and also patients and users of the health care system. During the development process feedback from members of the HLS_19_ Consortium was continuously collected. These included proven public health and/or health literacy experts in their respective countries with extensive research and/or policy experience. In addition, the views and perspectives of the relevant patient and user groups proved to be valuable. However, patient and user views could only be included from Germany, as the focus groups and pre-test were performed only in this country. A more extensive validation of the instrument in other countries is needed and will be possible in HLS_19_, since six more countries than Germany are planning to include this specific instrument also in their surveys (Austria, Belgium, Switzerland, France, Portugal, Slovenia). Furthermore, psychometric properties of the instrument need to be evaluated when population data are available.

In general, the broad approach taken in the item evaluation and finalization phase of the instrument allowed a variety of different perspectives to be incorporated. This proved to be fruitful in terms of patient and user participation, as well as for the applicability of the items to different country and system contexts. The instrument can thus be used not only in the context of HLS_19_, but also in other national and international population surveys.

Nevertheless, some limitations of the procedure also have to be mentioned. With the help of the scoping review, an overview of the existing definitions, concepts, and instruments could be obtained. However, since the object of the search encompassed a wide range of dimensions, levels, and labels it was difficult to find appropriate search terms. However, to minimize the risk of overlooking important publications, broad and general search terms were defined. This resulted in a high number of hits, of which most articles did not meet the inclusion criteria, after closer inspection. In addition, the concept mapping was mainly based on the identified literature, but the concept was further elaborated taking the developed definition of HL-NAV into account. Concept mapping workshops could not be carried out. However, the concept was intensively discussed and commented upon by experts of the HLS_19_ Consortium. A further restriction should be mentioned in terms of content. HL-NAV is primarily conceptualized with regard to the health care system and its socio-spatial environments because the demands on navigation are particularly high in this respect. However, there are also navigation requirements and difficulties in the area of disease prevention and health promotion. The same may apply to virtual and digital environments as well as in terms of communication and interaction: Certain expectations and rules are tied to the patient role, which is quite different from the role as client or consumer in the health care system. To find one’s way around these rules and norms, which have also changed considerably in recent years, navigation skills are also required. Thus, the transferability and further development of the concept to these and other relevant areas need further examination in the future.

## 5. Conclusions

The article describes the development of a new instrument to assess self-reported difficulties in processing information for navigating the health care system—the HL-NAV-HLS19. Thus, a topic is put on the agenda that—as this study demonstrates—has previously received little attention in health literacy research and instrument development.

Overall, the multi-step approach involving experts, stakeholders, and health care users enables a systematic and participatory development of the instrument in general populations in different countries. The integration of a professional perspective gives the opportunity to sharpen the contextual focus of the instrument. Key adjustments regarding comprehensibility and practicability were enabled by those who interact directly with the health care system as patients or users. The participation of international experts was considered mandatory, as the instrument is to be applied in an international context. These steps proved to be elaborate and time-consuming, and thus should be taken into consideration when planning new population surveys.

It can be assumed that a viable and feasible instrument has been developed that will generate comparable data on HL-NAV in different countries, for the first time, in the near future. Nevertheless, some caution should be made in terms of transferability because the validation steps to this point were only performed in Germany. Further research on the topic and the instrument is necessary and will be done with help of HLS_19_ data in seven countries.

## Figures and Tables

**Table 1:** Literature research findings.

**Reference**		**Definitions Related to HL-NAV**	**Provided Questionnaire**
1.	Rudd (2004) [41] (p. 19)	“The written word—in the form of signs, postings, and paper work […] often reflect the specialized language of the professionals who work there. Overall, the language, density, and complexity of these materials establish a literacy environment that makes demands on all who enter. [Navigating Hospitals] focuses on the literacy environment of hospitals and on the factors that hinder, as well as support, the ability of individuals to make their way to, and within, a hospital.”	/
2.	Rudd, Kirsch and Yamamoto (2004) [28] (p. 8)	“Finally, attention to barriers to programs, services, and care has shaped a fifth health literacy activity—one related to bureaucratic demands, referred to as “navigation.” Navigation of the health care system encompasses those activities related to rights and responsibilities, application for insurance and other coverage plans, and informed consent for procedures and studies.”	The Health Activities Literacy Scale (HALS)
3.	Osborne et al. (2013) [42] (p. 8)	“To find out about services and supports so they get all their needs met. Able to advocate on their own behalf at the system and service level.”	The Health Literacy Questionnaire (HLQ)
**Reference**		**Definitions Related to Navigation Competence**	**Provided Questionnaire**
4.	Paasche-Orlow and Wolf (2007) [43] (p. 20)	“A concept that has been advanced to explain how people with limited health literacy may have difficulty with using health services is the notion of navigation. The concept is meant to include all of the skills needed to go from one place to another in the pursuit of medical care.”	/
5.	Sofaer (2009) [9] (p. 76)	“The concept of “patient navigation” has been coined and used in a variety of ways and contexts. I use it here to denote the process(es) by which patients and/or their caregivers move into and through the multiple parts of the health care enterprise to gain access to and use its services in a manner that maximizes the likelihood of gaining the positive health outcomes available while minimizing inefficiencies that result, for both patients and providers, from complexity and poor coordination.”	/
6.	Perez et al. (2016) [44] (p. 1593)	“Navigation tasks can include successful medication recall, understanding copay and insurance requirements for medical visits, and knowing how to organize and respond to provider recommendations. Navigation and other self-management skills, […] depend on health literacy.”	Navigating Ability Questionnaire (NAV2)
7.	Fields et al. (2018) [45] (p. 482)	“The ability to locate and arrange services and supports for the care recipient.”	Health care communication and navigation of services and supports (HCNS)
8.	Gui, Chen and Pine (2018) [46] (p. 22)	“Navigational competence is a set of integrated capabilities consisting of knowledge and skills for individual health care consumers to go through complex service provision systems effectively.”	/
**Reference**		**Definitions Related to Interaction/Communication**	**Provided Questionnaire**
9.	Osborne et al. (2007) [47] (p. 197)	“Individual’s understanding of and ability to interact with a range of health organizations and health professionals, […] confidence and ability to communicate and negotiate with health care providers to get needs met.”	Health Education Impact Questionnaire (heiQ)
10.	Duke et al. (2015) [48] (p. 559)	“Confidence and ability to ask about and participate in treatment decisions.”	The Altarum Consumer Engagement (ACE) Measure

**Table 2:** Characteristics of focus group and pre-test participants.

Characteristics	Focus Group Participants (N = 32)		Pre-Test Participants (N = 33)	
	n	%	n	%
**Gender**				
male	16	50.0	16	48.5
female	16	50.0	17	51.5
**Age**				
18–29	7	21.9	8	24.2
30–44	14	43.8	7	21.2
45–59	5	15.6	9	27.3
60+	6	18.8	9	27.3
**School attainment**				
high school graduation	12	37.5	13	39.4
no high school graduation	20	62.5	20	60.6
**Employment status**				
employed	20	62.5	-	-
unemployed	7	21.8	-	-
retired	5	15.6	-	-
**Migration status**				
yes	8	25.0	-	-
no	24	75.0	-	-
**Chronic illness**				
yes	12	37.5	-	-
no	20	62.5	-	-
**Region ^a^**				
urban	16	50.0	-	-
more rural	16	50.0	-	-

^a^ refers only to the region where the focus groups took place.

**Table 3:** Final items classified by tasks and steps of information processing, I-CVI for initial Items and revision made.

HL-NAV Task	Step of Information Processing	Initial Item	I-CVI ^a,b^	Revision Made ^c^	Final Item
get an overview of the health care system, its structures, areas, functioning and type of health services	understand	Item 1	0.67	summarize items; add example	1. understand information on how the health care system works [e.g., which type of health services are available]
		Item 2	1.0		
get an overview of the health care system, its structures, areas, functioning and type of health services	appraise	Item 3	1.0	summarize items; revise wording	2. judge which type of health service you need in case of a health problem
		Item 4	0.83		
		Item 6	0.83		
be aware of health care costs and coverage	appraise	-	-	formulation of a new item with example	3. judge to what extent your health insurance covers a particular health service [e.g., are there any co-payments]
be aware of ongoing system changes and possible consequences for health care	understand	Item 15	0.67	revise wording; formulate more specifically	4. understand information on ongoing health care reforms that might affect your health care
be aware of rights and responsibilities	access	Item 10	1.0	revise wording	5. find out about your rights as a patient or user of the health care system
take decision concerning concrete services	apply	-	-	formulation of a new item with example	6. decide for a particular health service [e.g., choose from different hospitals]
find out about quality and judge if personal preferences are met	access	Item 7	1.0	revise wording	7. find information on the quality of a particular health service
find out about quality and judge if personal preferences are met	appraise	Item 8	1.0	formulate more specifically	8. judge if a particular health service will meet your expectations and wishes on health care
be aware of formal access requirements and rules of accessing and using health care	understand	Item 5	1.0	revise wording; formulate more specifically	9. understand how to get an appointment with a particular health service
find out about and use support options in order to navigate the health care system	access	-	-	formulation of a new item	10. find out about support options that may help you to orientate yourself in the health care system
find one’s way around a health care institution	apply	Item 13	0.83	summarize items; revise wording; add example	11. locate the right contact person for your concern within a health care institution [e.g., in a hospital]
		Item 14	1.0		
interact competently with health professionals and institutions in order to negotiate health care paths and settings	apply	Item 11	1.0	summarize items revise wording	12. stand up for yourself if your health care does not meet your needs
		Item 12	1.0		
be aware of rights and responsibilities	-	Item 9	0.67	delete	-
-	-	-	-	Add introduction	*“Now we would like to know how easy it is to inform yourself on finding your way around the health care system. It does not matter whether you use information for yourself or for someone else. By health service we typically mean a doctor, specialist, hospital, nursing home, rehabilitation or mental health facility.* *On a scale from very easy to very difficult, how easy would you say it is to”*

^a^ I-CVI = item-level content validity index. ^b^ S-CVI/Ave (content validity index for scale) for initial Item pool was 0.9. ^c^ The necessity for revisions was based on feedback from the expert and stakeholder evaluation, the focus group discussions and the pre-test as well as the feedback from the HLS_19_ Consortium.

## Appendix A

**Table A1:** Inclusion and exclusion criteria.

Criterion	Inclusion	Exclusion
Type of article	Original research articles Validation studies Empirical research Concept papers	Congress papers Study protocols Dissertations
Study focus	Providing an instrument measuring navigation/HL-NAV from patients perspective Providing a definition or conceptualizing navigation/HL-NAV Quantitative and qualitative	Intervention (navigation/HL-NAV is not the outcome) Professionals/organizations perspective
Language	English and German	Other
